# Highly Pathogenic H5N1 Influenza A Virus Strains Provoke Heterogeneous IFN-α/β Responses That Distinctively Affect Viral Propagation in Human Cells

**DOI:** 10.1371/journal.pone.0056659

**Published:** 2013-02-25

**Authors:** Markus Matthaei, Matthias Budt, Thorsten Wolff

**Affiliations:** Division of Influenza/Respiratory Viruses, Robert Koch-Institut, Berlin, Germany; University of Ottawa, Canada

## Abstract

The fatal transmissions of highly pathogenic avian influenza A viruses (IAV) of the H5N1 subtype to humans and high titer replication in the respiratory tract indicate that these pathogens can overcome the bird-to-human species barrier. While type I interferons (IFN-α/β) are well described to contribute to the species barrier of many zoonotic viruses, current data to the role of these antiviral cytokines during human H5N1 IAV infections is limited and contradictory. We hypothesized an important role for the IFN system in limiting productive infection of avian H5N1 strains in human cells. Hence, we examined IFN-α/β gene activation by different avian and human H5N1 isolates, if the IFN-α/β response restricts H5N1 growth and whether the different strains were equally capable to regulate the IFN-α/β system via their IFN-antagonistic NS1 proteins. Two human H5N1 isolates and a seasonal H3N2 strain propagated efficiently in human respiratory cells and induced little IFN-β, whereas three purely avian H5N1 strains were attenuated for replication and provoked higher IFN secretion. Replication of avian viruses was significantly enhanced on interferon-deficient cells, and exogenous IFN potently limited the growth of all strains in human cells. Moreover, IFN-α/β activation by all strains depended on retinoic acid-inducible gene I excluding principal differences in receptor activation between the different viruses. Interestingly, all H5N1 NS1 proteins suppressed IFN-α/β induction comparably well to the NS1 of seasonal IAV. Thus, our study shows that H5N1 strains are heterogeneous in their capacity to activate human cells in an NS1-independent manner. Our findings also suggest that H5N1 viruses need to acquire adaptive changes to circumvent strong IFN-α/β activation in human host cells. Since no single amino acid polymorphism could be associated with a respective high- or low induction phenotype we propose that the necessary adaptations to overcome the human IFN-α/β barrier involve mutations in multiple H5N1 genes.

## Introduction

Influenza A viruses (IAV) are the prototypic members of the *Orthomyxoviridae* and cause seasonal epidemic outbreaks of respiratory disease in humans with significant morbidity and mortality [1]. The epidemiology of human influenza is strongly influenced by a large natural reservoir of IAV in avian species. The segmented RNA genome of influenza viruses allows for reassortment of segments between human and avian IAV strains, which was elemental for the generation and introduction of pandemic IAV strains into the human population in 1957 and 1968 [2]–[4]. In contrast, direct transmission of avian IAV to humans *in toto* has been rarely noticed until the emergence of certain Asian H5N1 strains at the end of the last century [5], [6]. While this indicates the existence of a robust species barrier for avian IAV, it is still not completely understood which factors prevent efficient replication of avian influenza viruses in human hosts.

For a long period it was believed that the different receptors avian and human IAV recognize *via* the hemagglutinin (HA) to infect their respective target cells were the main reason for inefficient replication of avian IAV in humans. The HA of most avian strains recognizes terminal sialic acids with an α2,3-linkage expressed on the surface of avian cells, while human IAV prefer α2,6-linked sialic acids present on human tracheal cells [7]. However, human H5N1 isolates only rarely express HA proteins with adaptive changes facilitating binding of “human” receptor determinants [8]–[10]. In addition, α2,3-linked sialic acids were recently shown to be present especially in the lower human respiratory tract [11]–[15] indicating other factors to limit avian IAV replication in humans in addition to receptor specificity. Mutations in the viral polymerase increasing enzymatic activity and enabling replication at lower temperatures have then been identified as important requirements for adaptation [16], [17].

Remarkably, human H5N1 infections are rather rare in regard to the widespread exposure of humans to H5N1 viruses endemic in poultry [18]. On the other hand, high virus loads observed in H5N1 patients indicate that H5N1 viruses have the capacity to efficiently replicate in human hosts despite the presence of a vigorous cytokine response [19]–[22].

It is well established that seasonal influenza viruses activate the innate type I IFN response *via* viral 5′-triphosphorylated (5′-PPP)-RNAs, which are sensed by the cytoplasmic sensor RIG-I [23]–[25]. Subsequently, RIG-I signaling activates the expression of IFN-α/β genes *via* upregulation of the transcription factors IRF-3/−7, NF-κB and ATF-2/c-jun [26] and secreted IFN-α/β stimulates the expression of more than 100 latent genes, many of which encode factors with potent antiviral activity [27]. Type I interferon (IFN-α/β) induction, if not avoided or dealt with by the virus, leads to the establishment of an antiviral state in infected and bystander cells, which effectively suppresses virus replication [28]. Seasonal human influenza viruses achieve efficient replication by suppressing the activation of IFN-α/β genes *via* their NS1 protein, a pleiotropic factor that is abundantly expressed in infected cells [24], [25], [29]–[35]. Earlier studies of H5N1 infections of human lung epithelial cells and macrophages were inconclusive in this respect as they reported either weak [30] or strong induction [36]–[38] of IFN-α/β genes. This raised questions about the specific sensitivity of H5N1 strains to IFNs, whether H5N1 viruses trigger an alternative IFN induction pathway in human cells, and whether they possibly encode NS1 proteins with compromised functions.

The purpose of this study was to examine our hypothesis that the innate type I IFN response restricts the replication of H5N1 viruses in the human host and thereby contributes to the avian to human species barrier. Hence we compared a panel of available human and avian H5N1 strains with a prototypic seasonal IAV. Since the viral NS1 proteins are known as the main influenza viral regulator of the IFN-α/β response, we also put a special emphasis on the functionality and mode of action of H5N1 NS1 proteins in human cells.

Our analysis showed that two highly pathogenic human H5N1 isolates replicated at least as efficient as a prototypic seasonal H3N2 strain in different human epithelial cells and induced comparably low IFN-β secretion. In contrast, three analyzed avian H5N1 isolates activated a much more pronounced IFN-β secretion and were attenuated for replication indicating profound differences in their interaction with cellular innate defenses. Growth analyses of the H5N1 strains in IFN-deficient- and in IFN-α pre-conditioned cells demonstrated that the human type I IFN system potently reduces H5N1 virus growth. Viral IFN upregulation uniformly depended on the RIG-I sensor protein indicating that H5N1 viruses do not trigger an alternative IFN induction pathway. Reverse genetic analyses showed that all H5N1 NS1 proteins antagonize IFN induction similar as their counterpart of a prototypic seasonal strain. Hence we conclude that the IFN system contributes to the bird-to-human species barrier and suggest that efficient replication of H5N1 viruses in human cells requires adaptive changes to accommodate to the new host.

## Materials and Methods

### Cells and Viruses

Human A549 lung epithelial cells (ATCC CCL-185) and 293T cells (ATCC CRL-11268) were grown in Dulbeccòs modified eagle medium (D-MEM) supplemented with 10% heat-inactivated fetal bovine serum (FBS), 2 mM L-glutamine and antibiotics. Madin Darby canine kidney cells (MDCK) cells (ATCC CRL-2936) were cultivated in minimum essential medium (MEM) containing 10% FBS, glutamine and antibiotics and Vero cells were grown in serum-free Opti-Pro medium (Invitrogen). Unpolarized human bronchial epithelial Calu-3 cells (ATCC HTB-55) were grown in Eagle’s minimal essential medium as described, but 15% FBS were used [30]. Normal human bronchial epithelial (NHBE) cells (Lonza, Walkersville, USA) were grown as recommended by the manufacturer. Frozen stock cells were thawed, passaged once, seeded in 24-well-plates and used when nearly confluent. All cells were cultured in monolayers, maintained at 37°C and 5% CO**_2_**.

The two highly-pathogenic H5N1 influenza A virus strains A/Hong Kong/156/1997 (clade 0; obtained from the National Institute for Biological Standards and Control, Hertfordshire, UK) and A/Thailand/1 (KAN-1)/2004 (clade 1; obtained from P. Puthavathana, Bangkok, Thailand) were previously isolated from infected patients and are referred to as human H5N1 isolates throughout this report [39], [40]. The H5N1 strains A/chicken/Indonesia/R132/2004 (clade 2.1.1), A/duck/Vietnam/TG24-O1/2005 (clade 1) and A/common buzzard/Berlin/1/2006 (clade 2.2) were obtained from Brunhilde Schweiger (German National Reference Centre for Influenza at the Robert Koch-Institut, Berlin). All experiments and handling of samples containing H5N1 infectious particles were done in a biological safety level 3 facility. Stocks of human H5N1 isolates were grown in MDCK cells, while the avian isolates were propagated in 10 day old embryonated chicken eggs. The influenza A/Panama/2007/1999 virus (Pan/99) was grown in MDCK cells and used in its recombinant form as a prototypic seasonal influenza virus (see below).

### Generation of Recombinant Viruses

A recombinant eight plasmid system for the Pan/99 strain and respective constructs encoding the NS segments of H5N1 viruses were generated with the pHW2000 vector according to published procedures [41]. All plasmids were verified to contain the specific inserts with the expected sequence. The delNS1 construct was generated by reverse transcription from NS2-mRNA using an oligo-dT primer and amplification using the primers 5′-TAT TCG TCT CAG GGA GCA AAA GCA GGG TG-’3 and 5′-ATA TCG TCT CTT ATT AGT AGA AAC AAG GGT GTT TTT TAT TAA ATA AGC TGA AAT G-‘3 resulting in an insert encoding only the NS2 open reading frame flanked by the non-coding regions of the viral NS segment. Recombinant Pan/99 and mutant viruses were rescued by passaging cell culture supernatants of 293T cells transfected with the eight respective pHW2000-derived plasmids on Vero cells. The recombinant Pan/99 WT virus showed similar multi-cyclic growth and IFN-β induction on A549 cells compared to the natural isolate (data not shown).

### IFN-αA/D- treatment of Cultured Cells

A549 cells were treated with 500 IU/ml of human IFN-αA/D [*BglII*] (PBL) 6 hrs before infection and viral growth media was supplemented with the same IFN-α concentration. Infected cultures received fresh media containing IFN-α at each time-point of sample acquisition to maintain media volume and IFN-α concentration.

### Infections and Plaque Titrations

For infections, 80 to 90% confluent cell cultures were incubated with diluted stock virus for 45 min at room temperature. Infected cells were washed and incubated with D-MEM supplemented with 0.2% bovine albumin, 2 mM L-glutamine, 100 U/ml penicillin, 100 µg/ml streptomycin and 0.4 µg/ml (A549 cells) or 1.0 µg/ml of trypsin (MDCK, Vero cells). Plaque titer determination was done by infecting confluent layers of MDCK cells with serial dilutions of virus, which were then overlaid with 1.25% Avicel RC581 (FMC) in MEM supplemented with 0.2% BA, 0.05% NaHCO**_3_** and 0.01% DEAE dextran [42]. Plaques were visualized by either immunostaining of cells for viral nucleoprotein at 24 hrs p.i. (hpi) or by staining with crystal violet at 48 hpi. To stain for the IAV nucleoprotein, cells were permeabilized with 0.2% Triton X-100 and plaques were visualized on a Li-Cor Odyssey platform using a viral NP-specific monoclonal antibody (AbD Serotec Division of MorphoSys, clone AA5H) and a secondary anti-mouse IgG antibody (Li-Cor).

### IFN-β ELISA

Cell culture supernatant samples from infected cell cultures were stored at −80°C, thawed at room temperature and IFN-β concentrations were measured using a human IFN-β ELISA Kit (Fujirebio Inc./Invitrogen) according to the manufacturer’s instructions.

### Immunoblotting Analysis

Cells were lysed in Tris-HCl, pH 7.5 containing 1% NP40, 0.1% SDS, 2 mM EDTA, 10% glycerol, 137 mM NaCl and protease inhibitors. Protein content of lysates was estimated *via* Bradford assay (BioRad) and equal amounts of protein were separated by SDS gel electrophoresis and transferred to Protran**™** nitrocellulose membranes (Schleicher & Schüll, Germany). The following primary antibodies were used for antigen detection: mouse α-NP (AbD Serotec, clone AA5H), rabbit α-actin (Acris, NB600-532), rabbit α-RIG-I (Axxora, ALX-210-932) as well as the fluorophore-labeled secondary antibodies IRDye 800CW goat α-rabbit and IRDye 600 goat α-mouse (Li-Cor) or respective horseradish peroxidase-conjugated antibodies (Dako). Band intensities were quantified using the Li-Cor Odyssey software.

### siRNA Transfection Experiments

RIG-I-specific small interfering (si)RNA and non-target control siRNA used were previously described [25]. A549 cells seeded in 12-well plates were transfected with 150 ng siRNA and 6 µl HiPerfect (Qiagen, Hilden) according to the manufacturer’s protocol. 36 to 48 hrs after transfection, cells were infected with virus and IFN-β concentrations and knock-down efficiencies were determined by densitometric quantification of immunoblot blot bands from RIG-I and tubulin loading controls.

### IFN-β Promoter Luciferase Reporter Assay

293T cells were transfected in triplicates in poly-D-lysine coated 12-well plates using Lipofectamine2000 (Invitrogen). Cells were co-transfected with 1 µg of empty vector or pHW2000-NS plasmid, 50 ng of the IFN-β promoter reporter p125-luc and the pRL-TK-luc plasmid (Promega) to normalize transfection efficiency as described [25], [43]. 30 hrs post transfection cells were infected with Pan-delNS1 virus (MOI = 1) or mock infected. Cellular luciferase activities were determined 16 hrs p.i. using the Dual Luciferase Kit (Promega). The respective firefly luciferase activities were normalized and the increase in reporter activity in Pan-delNS1 infected cells compared to mock-infected cells was calculated.

### Statistical Analysis

Data is presented as mean +/− SEM if not indicated otherwise. The two-tailed student’s t-test was used to delineate significant differences between data points using the Prism6 software (Graph Pad), p≤0.05 was considered significant. Significance is depicted as * for p≤0.05, ** for p≤0.01 and *** for p≤0.001, respectively.

### Sequences

The hitherto unpublished sequences for A/Common buzzard/Berlin/1/2006 (EPI349049-52, EPI346565, EPI346566, and EPI346568) and A/Chicken/Indonesia/R132 (EPI354072–78 and EPI354080) were submitted to the GISAID database (http://gisaid.org) and complete sequence information for A/Panama/2007/99 (DQ487333-40) was deposited at Genbank (http://www.ncbi.nlm.nih.gov). Sequence information of the remaining H5N1 strains can be accessed in GISAID (A/Duck/Vietnam/TG24/05, EPI_ISL_70397) and Genbank (A/Hong Kong/156/97 and A/Thailand/1 (KAN-1)/2004).

## Results

### Replication of Human and Avian Influenza H5N1 Virus Isolates in Human Lung Epithelial Cells

We compared five closely related influenza A virus isolates of the H5N1 subtype with the prototypic seasonal H3N2 strain A/Panama/2007/99 (Pan/99) for viral growth and activation of the type I IFN system in human cells. The H5N1 strains A/Hong Kong/156/97 (HK/97) and A/Thailand/1(Kan-1)/2004 (Thai/04) were derived from lethally infected human patients, whereas A/chicken/Indonesia/R132/2004 (Ch/Ind), A/duck/Vietnam/TG24-O1/2005 (Duck/VN) and A/common buzzard/Berlin/1/2006 (Buzz/Bln) were of avian origin. Since we analyzed the contribution of the viral NS1 protein in the control of type I IFN induction, we also included a delNS1 deletion variant of the Pan/99 (H3N2) virus lacking the NS1 coding region in our study [44].

**Figure 1:**
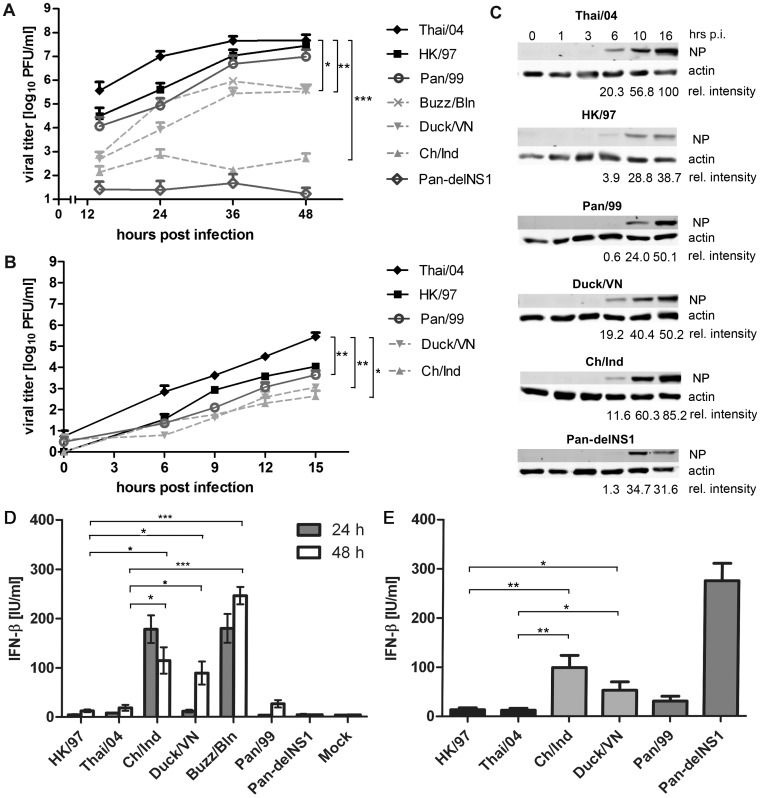
Replication and IFN-β induction by human and avian H5N1 strains in human cells. (A, B) A549 cells were infected at a multiplicity of 0.01 (A) or 1 (B) with the highly-pathogenic human H5N1 IAV isolates A/Hong Kong/156/1997 (HK/97) and A/Thailand/1 (KAN-1)/2004 (Thai/04), the avian H5N1 strains A/chicken/Indonesia/R132/2004 (Ch/Ind), A/duck/Vietnam/TG24-O1/2005 (Duck/VN) and A/common buzzard/Berlin/1/2006 (Buzz/Bln) as well as with the prototypic seasonal A/Panama/2007/99 (Pan/99) H3N2 virus and its mutant variant with a deleted NS1 gene (Pan-delNS1; shown only in panel A). Supernatant of infected cell cultures was sampled at the indicated time points and viral titers were determined by plaque assay. Mean data+SEM of at least three independent experiments is shown. (C) Immunoblot analysis of lysates from A549 cells infected at MOI = 1 using a viral NP-specific antibody at the indicated time points post infection (p.i.). Relative intensities of NP bands normalized to actin controls are depicted in comparison to Thai/04, which was arbitrarily set as 100%. (D, E) Concentrations of IFN-β in cell culture supernatants of A549 cells infected with the indicated viruses at low (MOI = 0,01, panel D) or high multiplicity (MOI = 1, panel E) determined *via* ELISA at 24 and 48 hpi (D) or at 16 hpi (E). Mean data of at least three independent experiments +/– SEM is shown. Symbols indicate significant differences between given results with p<0.05 (*), p<0.01 (**), or p<0.001(***), respectively, as determined by unpaired two-tailed t-tests.

The two human H5N1 isolates reached approximately four times higher titers during multi-cyclic growth on human A549 lung epithelial cells than the seasonal H3N2 strain at 48 hrs p.i. (Figure 1A). In contrast, growth of the three avian H5N1 isolates was considerably lower in the range of one (Duck/VN, Buzz/Bln) to four (Ch/Ind) orders of magnitude, respectively. The Pan-delNS1 virus did not replicate in these cells demonstrating the essential contribution of NS1 to efficient viral growth, which is consistent with previous findings for delNS1 derivatives of the mouse-adapted strains A/PR/8/34 (H1N1) and A/SC35M (H7N7) [29], [45]. Replication of avian H5N1 viruses was also attenuated in human bronchiolar Calu-3 cells and primary bronchiolar epithelial cells by one or more logs compared to the human H5N1 isolates, suggesting a generally compromised capacity of the avian strains to replicate in human cells (Figure S1A and B). Single-cycle growth analysis confirmed the compromised growth of the avian H5N1 strains in human cells, since the human H5N1 isolates also replicated considerably faster, with the Thai/04 isolate reaching titers three orders of magnitude higher than the avian H5N1 isolates at 15 hrs p.i. (Figure 1B). The poor growth of the avian H5N1 isolates was most likely not caused by a generally low rate of primary infection, since the amounts of viral nucleoprotein (NP) detected in cells infected with avian strains were comparable or even higher than in cells infected with human isolates (Figure 1C).

### Differential Induction of IFN-β of Human and Avian Influenza H5N1 Virus Isolates

Analyzing the accumulation of type I IFN in the supernatants of infected A549 cells, we noticed that the poorly growing avian H5N1 strains induced an up to 15 times stronger IFN-β secretion from human cells than the human H5N1 and seasonal viruses, regardless whether infection was initiated at low (24 and 48 hpi) or high (16 hpi) multiplicity (Figure 1D and E). Conversely, there were only minor differences in the low IFN-β inductions between the well growing human isolates (HK/97 and Thai/04) and the seasonal Pan/99 strain. The Pan-delNS1 mutant virus strongly induced IFN-β when applied at high MOI (Figure 1E), but failed to do so after low multiplicity infection (Figure 1D), most likely due to its inefficient replication (Figure 1A). Of note, we also evaluated the human and avian virus strains for their capacity to stimulate IFN-β secretion from primary monocyte-derived macrophages and human bronchiolar Calu-3 cells, and noticed similar ratios of IFN secretion as were observed for A549 cells infected at high MOI (Figure S2 and data not shown). These findings indicated that H5N1 influenza viruses vary in their capacity to inhibit and/or to induce antiviral IFN-β in human cells and suggested that IFN hyper-induction can cause or contribute to the attenuated propagation of the avian H5N1 isolates.

### H5N1 Virus Growth is Sensitive to IFN-α/β

**Figure 2:**
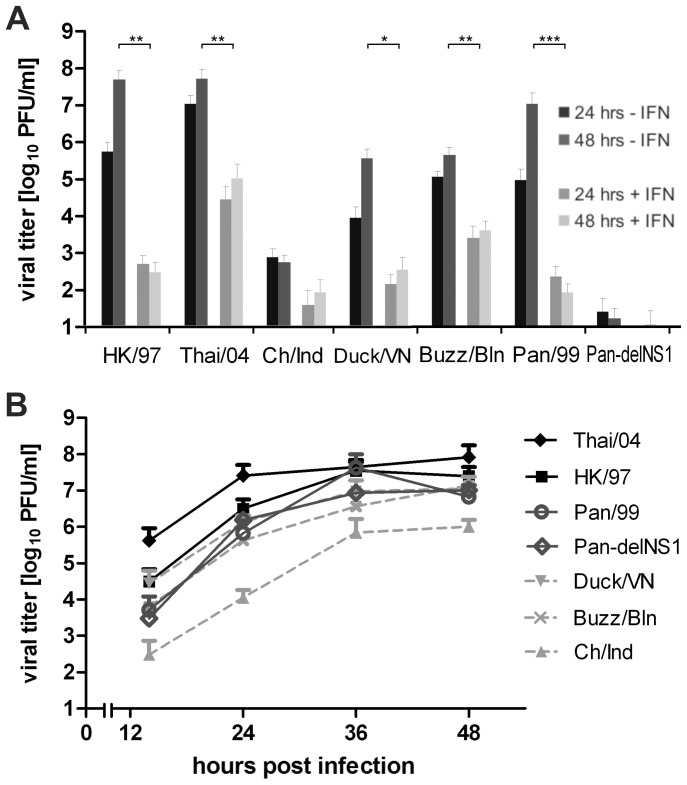
H5N1 virus growth is sensitive to IFN-α/β. (A) A549 lung epithelial cells were treated with 500 U/ml recombinant IFN-α before and during infection with the indicated virus strains (MOI = 0.01). Viral titers were determined by plaque assay. Mean titers at 24 and 48 hours post infection of at least 3 independent experiments+SEM are presented. For comparison, the graph also depicts virus titers determined in untreated A549 cells at 24 and 48 hpi. shown in Figure 1A. Symbols indicate significant differences between given individual results with p<0.05 (*) or p<0.01 (**), respectively. (B) Virus growth of human and avian H5N1 isolates, Pan/99 (H3N2) and its deltaNS1 variant on IFN-α/β-deficient Vero cells infected at a MOI of 0.01. Samples of the infected cell cultures supernatants were taken at the indicated time-points and viral titers were determined by plaque assay (mean data+SEM; N ≥3).

To examine if there was a causal relationship between strong IFN-β secretion and low replication of some H5N1 strains, we studied their replication in A549 cells pre-conditioned and treated with 500 IU/ml IFN-α throughout the experiment. IFN treatment strongly reduced the growth of all human and avian strains up to five orders of magnitude compared to non-treated cells (Figure 2A). The human Thai/04 (H5N1) strain still reached the highest titers among the investigated strains. Thus, IFN-α/β potently restricted H5N1 virus growth in human cells and its strong induction by the avian H5N1 viruses is likely to compromise their growth on human lung cells.

To further validate that the IFN system does restrict H5N1 growth, we also examined viral propagation on Vero cells that lack functional IFN-α/β genes [46], [47] and expected enhanced growth especially of the strongly IFN inducing strains. In fact, the replication of the Pan-delNS1 mutant virus in these IFN-deficient cells was increased by six logs compared to A549 cells (compare Figures 2B and 1A), highlighting the importance of NS1-mediated suppression of the innate antiviral defense [48]. The human H5N1 strains and the Pan/99 WT virus reached similar titers in both cell lines, indicating their capacity to replicate efficiently in an interferon-competent host cell. Interestingly, in Vero cells the replication of the avian strains was much more similar to that of the human isolates. While in A549 cells at 48 hrs p.i. the titers for avian strains Ch/Ind, Duck/VN and Buzzard/Bln were approximately 52.000-, 81- and 69-fold lower than that of the human HK/97 strain (Figure 1A), these differences were reduced to 25-, 2- and 2-fold in Vero cells, respectively (Figure 2B).

### The IFN-α/β System is Activated via RIG-I during H5N1 Infection

An explanation for the higher levels to which the avian H5N1 isolates induced IFN-β secretion in human cells could be that they stimulate IFN-α/β induction in a different manner than human IAV, which are known to trigger IFN-α/β secretion *via* the RNA helicase RIG-I and to suppress this pathway via their NS1 proteins [25], [49]. We hence considered three different explanations for the more extensive induction of IFN-β by the avian H5N1 strains in our analysis: First, activation of a cellular receptor governing an alternative or additional IFN-α/β inducing pathway not blocked by the viral NS1 protein. Second, an insufficient inhibition of RIG-I signaling by the “avian” NS1 proteins, or third, a hyperstimulation of RIG-I signaling during avian H5N1 infections.

**Figure 3:**
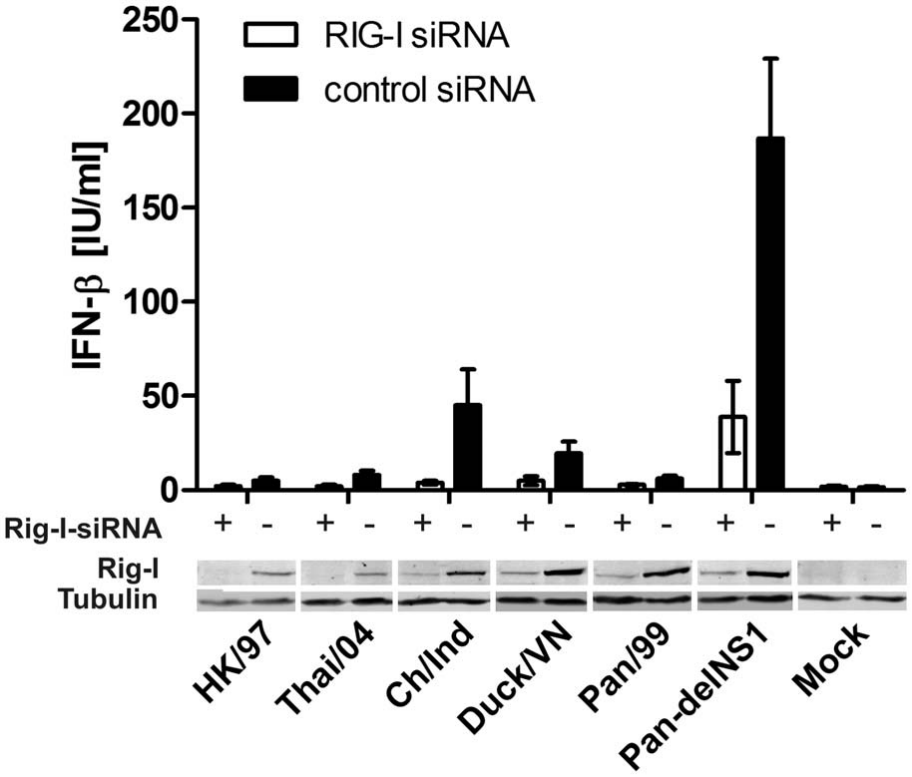
IFN-α/β secretion in response to H5N1 infection depends on RIG-I expression. A549 cells, transfected with either RIG-I-specific (+, open columns) or control (–, solid columns) siRNA were infected with the indicated H5N1 and the Pan/99 (H3N2) derived viruses (MOI = 1). IFN-β concentrations were determined *via* ELISA (Fujirebio, Japan) in cell culture supernatants taken 16 h post infection (upper panel) and RIG-I expression was determined by immunoblotting (lower panel). Mean data +/– SEM of four independent experiments and a representative immunoblot is shown.

To test whether H5N1 isolates might induce IFN-α/β via a different pathway, we explored the consequences of siRNA-mediated knock-down of RIG-I in A549 cells on IFN-α/β induction during H5N1 virus infection. Immunoblot analysis of siRNA-treated cells confirmed a reduction of RIG-I protein levels by 85% on average (Figure 3, lower panel) as determined by densitometric analysis. Expression of other RNA sensors like MDA5, DDX1 and TLR3 was not affected by siRNA treatment (data not shown). Significantly, cells transfected with the RIG-I-specific siRNA secreted uniformly about 80% less IFN-β after infection with each of the viruses compared to control siRNA transfected cells (Figure 3) (p = 0.0411, exact p-value, two-tailed Mann-Whitney test). The strong decrease in IFN-β accumulation in supernatants of cells with little detectable RIG-I, suggests that other, RIG-I-independent, signaling pathways do not contribute significantly to the induction of IFN-α/β during avian H5N1 virus infections in human cells.

### The H5N1 NS Segments Complement the Growth of a Seasonal H3N2 Virus in Human Cells and Encode Functional IFN Antagonists

**Figure 4:**
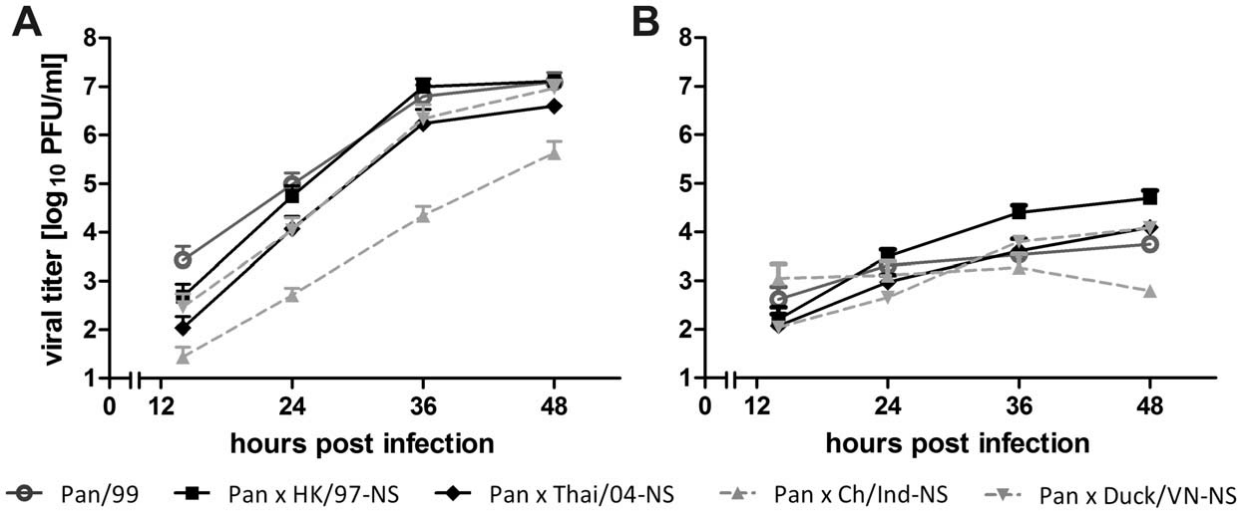
Replication of reassortant Pan/99 (H3N2)×H5N1-NS viruses in lung epithelial cells. (A) A549 cells were infected with the indicated Pan/99 (H3N2) wild-type and reassortant viruses carrying a H5N1-NS segment (MOI = 0.01). Aliquots of the supernatants were taken at the indicated time points and viral titers were determined by plaque titration. Mean data of at least two independent experiments+SEM are shown. (B) Virus growth on IFN-α treated A549 cells (500 IU/ml, 6 h before infection (MOI = 0.01) and during virus growth) was determined as described in panel A. Shown are the average results of two independent experiments+SEM.

Next, we examined the capacity of the H5N1 NS1 proteins to inhibit the RIG-I mediated induction of IFN-β in human cells. All five NS1 proteins share less than 80% sequence identity on the amino acid level with their counterpart of the seasonal Pan/99 virus. Among the H5N1 NS1 homologs, the HK/97 differed by up to 13% from the four other strains, which in turn differed by less than 6% from each other. Thus, we generated and characterized 7+1 reassortant viruses that carry the respective H5N1-NS gene segments in the background of the seasonal Pan/99 strain (H3N2). Interestingly, the heterologous H5N1-NS segments complemented the growth of the H3N2 reassortants in human A549 cells to a similar level as was observed for the parental Pan/99 virus. Only the Pan/99×Ch/Ind-NS reassortant was attenuated to some extent (Figure 4A), but still rescued the replication of the reassortant by more than 4 orders of magnitutde compared to the NS1-deleted Pan/99 mutant virus (Figure 1A). Thus, the efficient growth of the reassortant viruses indicated that the encoded H5N1-NS1 proteins function well in human cells.

We also examined the propagation of these reassortant viruses in IFN-α-treated cells, since H5N1 NS1 proteins had previously been reported to interfere with IFN signaling and enhance virus replication [50]. However, the growth of all reassortants was strongly attenuated by IFN treatment (Figure 4B) as had also been observed for the parental viruses (Figure 2A).

**Figure 5:**
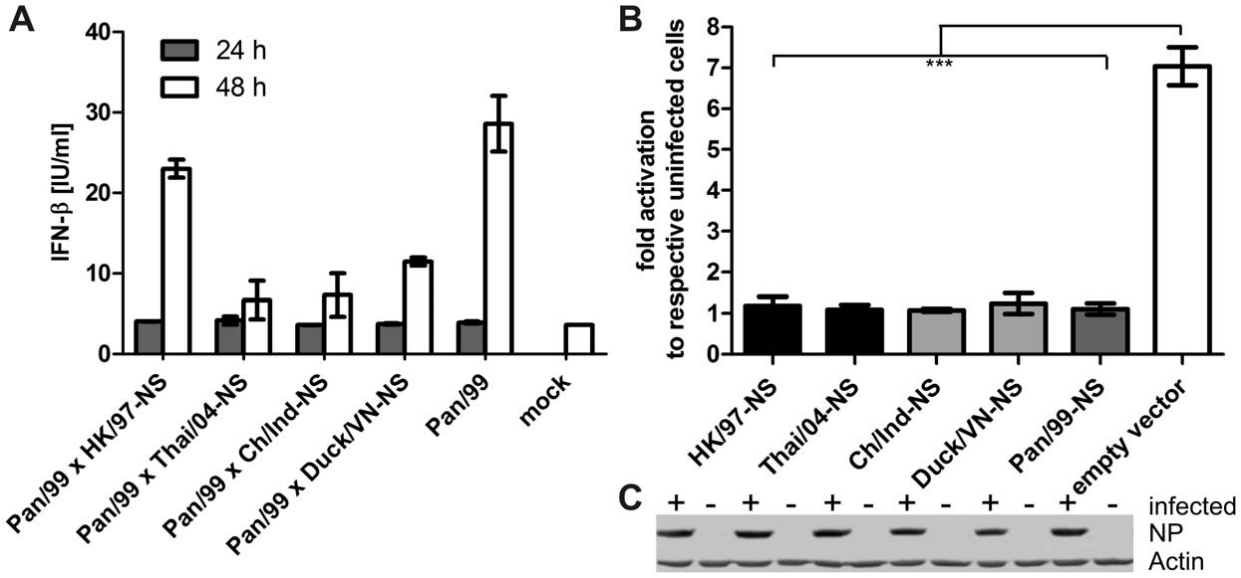
H5N1 NS1 proteins inhibit the induction of type I IFN in human cells. (A) IFN-β concentrations of supernatants sampled from A549 cell cultures infected with the Pan99 WT and different NS reassortant viruses (H3N2) (MOI = 0.01) at 24 and 48 hrs p.i., (N≥2+/–SEM). (B) Human 293T cells were co-transfected with plasmids expressing the H5N1- or Pan/99 (H3N2)-derived NS segments or empty vector, the human IFN-β promoter reporter plasmid p125-Luc and pRL-TK-Luc to control for transfection efficiency. Subsequently, cells were infected for 16 h with Pan-delNS1 to stimulate the IFN-β promoter before luciferase reporter activity was determined in cell lysates. IFN-β promoter activation in infected cells is shown as x-fold stimulation of firefly luciferase activity compared to transfected and mock infected cells. Mean data of three independent experiments is shown +/– SD. (C) Immunblot detection of viral NP after Pan-delNS1 (H3N2) infection confirmed equal stimulation of the transfected cells analyzed in panel B.

To further explore the IFN antagonistic activity of the different H5N1 NS1 proteins, we quantified IFN-β release from human cells infected with the reassortant viruses. This analysis showed that the reassortants induced equally low (Pan/99×HK/97-NS) or even less IFN-β secretion compared to the Pan/99 WT virus (Figure 5A), which was consistent with their efficient growth in A549 cells (Figure 4A). Hence, the reduced growth of the Pan/99×Ch/Ind-NS reassortant virus (Figure 4A) resulted most likely from a reduced compatibility between the Ch/Ind NS- and residual Pan/99 proteins, but not from a stronger type I IFN induction.

We also employed an established reporter assay to examine the suppression of viral IFN-beta promoter activation by the H5N1 NS1 proteins [25]. 293T cells were co-transfected with an IFN-β promoter reporter and NS1 expression plasmids or empty vector. Subsequently, cells were infected with the Pan-delNS1 virus for 16 hrs before reporter activity was determined. The Pan-delNS1 virus increased IFN-β promoter activity by 8-fold, whereas the reporter was hardly activated in cells expressing the respective NS1 proteins regardless of whether they were derived from a human or an avian virus strain (Figure 5B). Equal infection of the transfected A549 cells by the Pan/99-delNS1 mutant virus was verified by immunoblot detection of the viral NP (Figure 5C). Collectively, these findings demonstrate that NS1 proteins encoded by either human or avian H5N1 strains efficiently antagonize the induction of the IFN-α/β system in human lung cells.

### Comparison of the H5N1 Influenza Virus Backgrounds

The results indicated so far that differential activation of the IFN-α/β system by the panel of H5N1 viruses was neither influenced by a RIG-I-independent signaling pathway nor by a failure of some NS1 proteins to silence IFN-α/β gene expression. Thus, other viral signatures appear to be responsible for the observed strain-specific growth- and IFN induction phenotypes. To discover a putative underlying genetic element(s) we sequenced the examined H5N1 isolates and screened them for genetic polymorphism(s) previously described to be involved in H5N1 adaptation to mammalian hosts and/or pathogenicity [51]–[56]. In Table 1, these specific amino acid positions in the viral proteins previously linked with viral pathogenesis and/or adaptation to mammalian hosts are summarized for the different virus strains. Unfortunately, none of those specific amino acid constellations alone such as the PB2 polymorphisms E627K or D701N could explain the differences observed between the avian and human strains as these did not display common phenotype-specific variations. In fact, the three analyzed avian H5N1 strains have only two differences to the human strains in common: Ile-168 of the M1 protein and Thr-127 of the NS1 protein (Table 2). Thr-168 in the M1 protein of the human H5N1 isolates is unlikely to reflect an adaptive change as 30% of avian and 50% of human H5N1 isolates have a Thr residue at this position. Interestingly, NS1 amino acid 127 of seasonal IAV has been described to regulate the antiviral kinase PKR [57]. However, we did not observe differences in the activation of PKR by human or avian H5N1 strains (data not shown). Hence, the different phenotypes observed are presumably caused by the interplay of strain-specific polymorphisms and are possibly encoded in more than one single viral gene.

**Table 1:** Differences in H5N1 influenza virus proteins*.

Virus strain	PA		PB1	PB1-F2	PB2					
	127	336	317	66	271	318	355	627	701	702
**A/Hong Kong/156/1997**	V	L	**I**	N	T	R	**K**	E	D	K
**A/Thailand/1 (Kan-1)/2004**	V	L	M	N	T	R	R	E	**N**	K
**A/chicken/Indonesia/R132/2006**	V	L	M	N	T	R	R	E	D	K
**A/duck/VietNam/TG24-01/2005**	V	L	M	N	T	R	R	E	D	K
**A/common buzzard/Berlin/1/2006**	V	L	M	N	T	R	R	**K**	D	K
**A/Panama/2007/1999**	V	L	M	N	**A**	R	R	**K**	D	R
	**NS1**								**NS2**	
	**42**	**Δ80–84**	**92**	**103**	**106**	**189**	**195**	**228**	**31**	**56**
**A/Hong Kong/156/1997**	**S**	−	**E**	**L**	**I**	D	S	**P**	M	H
**A/Thailand/1 (Kan-1)/2004**	**S**	+	D	F	M	D	**T**	S	M	H
**A/chicken/Indonesia/R132/2006**	**S**	+	D	F	M	D	S	S	M	H
**A/duck/VietNam/TG24-01/2005**	**S**	+	D	F	M	D	**T**	S	M	H
**A/common buzzard/Berlin/1/2006**	**S**	+	D	F	M	D	S	S	M	H
**A/Panama/2007/1999**	**S**	−	D	F	M	D	S	S	M	H
	**HA1**							**NA**		
	**86**	**124**	**138**	**156**	**212**	**228**	**263**	**ΔStalk**		
**A/Hong Kong/156/1997**	A	N	**L**	A	**E**	G	**T**	+		
**A/Thailand/1 (Kan-1)/2004**	**V**	**S**	Q	**T**	**R**	G	**T**	+		
**A/chicken/Indonesia/R132/2006**	A	D	**L**	A	K	G	A	+		
**A/duck/VietNam/TG24-01/2005**	**V**	**S**	Q	**T**	**R**	G	**T**	+		
**A/common buzzard/Berlin/1/2006**	A	D	Q	A	K	G	**T**	+		
**A/Panama/2007/1999**	A	−	R	K	N	S	S	−		

* Shown are amino acids at positions associated with high pathogenicity or replication competence of H5N1 viruses in mammals. Amino acids described to confer high pathogenicity/virulence are shown in bold [51]–[57], [79]–[82]; summarized in: [101]. No relevant positions were noticed in NP, and parts of the human H5N1 isolates’ PB2 and NP sequences were taken from public databases.

**Table 2:** Common differences between examined human and avian H5N1 strains*.

	*NS1*	*M*
Virus strain	*127*	*168*
A/Hong Kong/156/1997	D	T
A/Thailand/1 (Kan-1)/2004	V	T
A/chicken/Indonesia/R132/2006	T	I
A/duck/Vietnam/TG24-01/2005	T	I
A/common buzzard/Berlin/1/2006	T	I
A/Panama/2007/1999	N	T

* Shown are the only two amino acid positions in which the examined avian H5N1 isolates commonly differ to the human H5N1 strains.

In conclusion, our study revealed that the ability to prevent or avoid induction of the human type I IFN system substantially contributes to enhanced replication of H5N1 viruses. This finding is corroborated by the inverse correlation of strong IFN-β induction with reduced replication among the human and avian H5N1 strains. Surprisingly, all H5N1 NS1 proteins functioned as effective IFN antagonists in infected and transfected cells indicating that not a limitation in avian NS1 activity but rather an exceptionally strong IFN induction by the tested avian strains restricts viral replication in human cells.

## Discussion

Highly pathogenic H5N1 strains of the Asian lineage circulating in birds since 2003 are unique as they cause few but regular human infections and a case fatality of almost 60% [58], while most other avian influenza viruses are not known to infect humans. Given the recent indications for the occurrence of mild or even asymptomatic H5N1 infections, it is unclear whether all or only a fraction of the circulating H5N1 strains have this high virulence potential in humans as even highly related virus isolates differ considerably in their pathogenicity in mammalian species like mice and ferrets [59]–[61]. Since human airways express both α2,6- and α2,3- linked receptors [13] and therefore receptor specificity is unlikely to completely explain the severe outcomes of H5N1 infections in humans, we examined a potential contribution of the human type I IFN system to the bird-to-human species barrier. Special emphasis was put on the activation and suppression of the human type I IFN system by H5N1 viruses since many examples have shown that innate immune responses restrict the replication of RNA viruses in a new host [62]–[66] and we wondered whether this is also the case for avian and/or human H5N1 isolates.

We observed, that the growth of different H5N1 isolates from birds or humans was significantly reduced on IFN-α pretreated cells which is in line with and extends previous reports showing that type I IFNs have a clear antiviral effect against H5N1 viruses in several mammalian species [18], [30], [50], [67]–[70]. Interestingly, at least two of three avian isolates examined induced an enhanced secretion of IFN-β from human epithelial cells and showed an attenuated growth, while a seasonal H3N2 strain and the two human H5N1 isolates induced only little IFN secretion and replicated efficiently (Figure 1). Although it is tempting to speculate that low IFN induction is a distinctive feature of H5N1 patient isolates, we are aware that additional analyses are required to validate this conclusion. Replication of the avian isolates but not the human strains was strongly increased on IFN deficient primate Vero cells (Figure 2B), which is in line with a role of the type I IFN system to limit their growth in IFN-competent cells. Hence, our study revealed a significant contribution of the type I IFN system to limit H5N1 replication in human cells. Enhanced IFN-α/β responses in human cells have also been reported for avian H4, H7 and H12 subtype viruses [71], [72] and may indicate a common feature of avian IAV in human cells. Interestingly, the human H5N1 isolates and the seasonal H3N2 strain showed similarly efficient growth and provoked only a modest type I IFN response in human lung cells and macrophages, which agrees with an earlier report [30], but seemingly differs from others [36], [37]. However, these apparent differences in the outcomes might be explained retrospectively by strain specific differences in regard to the H5N1 strains analyzed and the respective H1N1 and H3N2 seasonal control strains [73], [74].

The heterogeneous phenotypes of the examined human and avian H5N1 viruses concerning growth and IFN control in human cells, prompted us to examine possible mechanistic explanations for this phenomenon. We and others have previously established that seasonal IAV infections are recognized *via* RIG-I that is regulated by the viral NS1 protein [25], [30], [32], [49]. However, it had not been excluded that H5N1 viruses stimulate an additional or alternative IFN inducing signaling pathway such as the ones governed by the cellular MDA5, DDX1, TLR3 or TLR8/9 receptors [75], [76]. Still, our analysis showed that human RIG-I functions also as the major sensor for avian H5N1 viruses since its knock-down almost eliminated IFN-β production in infected epithelial cells (Figure 3). This confirms and extents a similar recent finding for H5N1 virus of the outbreak in Hong Kong in 1997 [77]. Interestingly, the phenotypes of Pan/99-derived 7+1 reassortant viruses expressing heterologous NS segments indicated that the NS1 proteins of both avian und human H5N1 isolates promoted virus replication (Figure 4) and suppressed activation of the human IFN-β promoter (Figure 5) in human cells. The latter aspect is reminiscent to a study of divergent NS1 proteins from unrelated avian viruses which also effectively antagonized the human type I IFN system [71]. Hence, we have to reject the possibility that differences in the NS1 proteins explain why some H5N1 strains replicated well in the human host, while others did not. This is in contrast to the findings of a recent study showing that mutations in the NS1 protein of a H3N2 subtype virus contribute to enhanced IFN antagonism and replication in mammalian cells [78]. Rather than NS1, our results suggest the involvement of other viral factors, in which allelic differences generated before or during virus transmission from an avian to a human host contribute to the low IFN inducing potential of the examined human H5N1 strains, in contrast to the avian isolates.

Previous comparisons of closely related H5N1 isolates with high and low pathogenicity identified a number of amino acid polymorphisms in the HA, polymerase and NS genes that increase viral virulence and/or replication in mammalian hosts (Table 1) [51], [54], [78]–[82]. To explain the abundant IFN induction by some H5N1 strains despite expression of functional NS1 proteins, we consider two possibilities: First, the viral polymerase synthesizes full-length genomic viral 5`-PPP RNAs and shorter versions thereof that stimulate RIG-I in infected cells [24], [83], [84]. Thus, we speculate that strong viral IFN-β induction could be caused by an excess production of 5′-PPP-RNAs that overwhelms the inhibition by viral antagonistic proteins in infected human cells. Accordingly, increased polymerase activity and IFN-β induction correlated in a study with chimeric viruses expressing the PA subunit of an H5N1 virus [85]. Our amino acid alignments failed to associate a single genetic determinant in the viral polymerase genes with this phenotype. However, identification of the responsible element(s) is likely to be a complex task, since the activity of influenza virus polymerase in mammalian cells is not only controlled by polymorphic amino acids such as the ones found at positions 701 or 627 in PB2 [19], [86]–[88]. Rather, the levels of viral 5′-PPP-RNAs synthesized in infected cells are also regulated by the viral NEP [89], [90] indicating that the kinetic, stability and level of NEP expression are important elements of polymerase regulation, which are poorly defined yet. Second, control of RIG-I-dependent signals by IAV is more complex than was previously appreciated. Recent analyses showed that not only NS1, but also the viral RNA polymerase subunits (PB2, PB1 and PA) and the PB1-F2 proteins regulate IFN induction, possibly by interactions with the mitochondrial MAVS/IPS receptor protein for activated RIG-I [91]–[94]. It is therefore possible that (an) uncharacterized polymorphism(s) in one or more of the respective genes determines or contributes to the high- and low IFN induction phenotypes of H5N1 strains. Even sequence comparisons of the two most potent IFN-β inducing strains Buzz/Bln and Ch/Ind with the human H5N1 isolates did not reveal any known polymorphic position(s) associated with a high virulence or IFN-β inducing phenotype. Both avian strains differed from the human H5N1 viruses in the HA1 (124, 212), PA (653), PB2 (339) and NA (363, 435) proteins in addition to the previously discussed positions in M1 and NS1. The differences in the HA proteins presumably reflect that these viruses belong to different clades [95] and the variations G363N and S435G in NA and Thr339Lys as in PB2 are common to both avian and human H5N1 isolates [96]. Amino acid 653 in the PA protein (S in both avian isolates instead of P in the human H5N1 strains) is part of an α-helical region described to be important for the interaction with PB1. The mutations E656A und G657A are known to reduce this interaction resulting in reduced transcription and replication of IAV [97], [98]. To determine if those or other individual differences in the polymerase or other proteins encoded by human and avian H5N1 isolates cause the different levels of IFN-α/β induction will be part of future work. Future work may also clarify, whether the human H5N1 isolates acquired their adaptation(s) during initial replication in the human host [99], or if they were randomly acquired in birds as a prerequisite for transmission into humans, which would foster our ability to assess the zoonotic threat by H5N1 strains [100].

## Supporting Information

Figure S1 **H5N1 virus replication in human Calu-3 and normal human bronchial epithelial (NHBE) cells.** Virus growth on Calu-3 (A, MOI 0.01) and NHBE (B, MOI 1) cells was analyzed *via* plaque titration of samples of cell culture supernatants taken at the indicated time points after infection (N≥2,+SEM).
(TIF)

Figure S2 **IFN-β secretion of influenza virus-infected human monocyte derived macrophages.** (A) Monocytic cells were isolated from buffy coats of healthy human blood donors and were differentiated in vitro. The resulting cultured cells are shown to express the typical markers for macrophages via antibody staining and FACS analysis. The prominent population of cells in the forward/sideward scattering (>90% of the cells, data not shown) expresses CD206, CD14, HLA-DR and little CD86. Black lines indicate the number of cells with a specific signal intensity of the used antibodies, grey lines represent isotype antibody controls. (B) Monocyte-derived human macrophages were infected for 24 h (MOI = 2) with the human and avian H5N1 strains, Pan/99 (H3N2) and its mutant variant with a deleted NS1 gene. IFN-β concentrations of cell culture supernatants were determined via a bead-based cytokine assay (Panomics). Shown are mean IFN-β concentrations +/− SEM of macrophage cultures obtained from three different donors independently infected in triplicates.
(TIF)

Methods S1 **Preparation and infection of monocyte-derived macrophages and FACS analysis.**
(DOCX)
