# Supplementary figures and images for: Highly Pathogenic H5N1 Influenza A Virus Strains Provoke Heterogeneous IFN-α/β Responses That Distinctively Affect Viral Propagation in Human Cells
Source: PLoS One. 2013 Feb 25;8(2):e56659. doi: 10.1371/journal.pone.0056659 (PMC3581526; doi:10.1371/journal.pone.0056659)

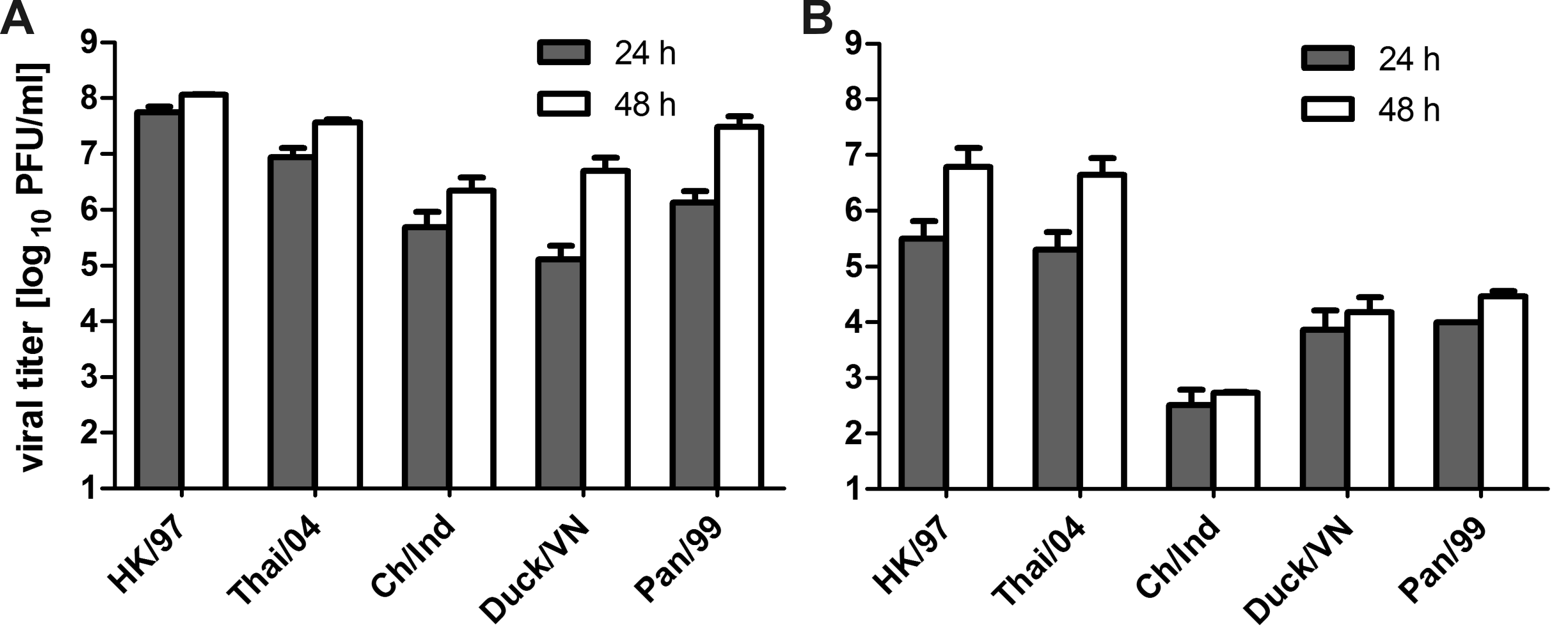

Supplement: Figure S1 — H5N1 virus replication in human Calu-3 and normal human bronchial epithelial (NHBE) cells. Virus growth on Calu-3 (A, MOI 0.01) and NHBE (B, MOI 1) cells was analyzed via plaque titration of samples of cell culture supernatants taken at the indicated time points after infection (N≥2,+SEM). (TIF) [file pone.0056659.s001.tif]

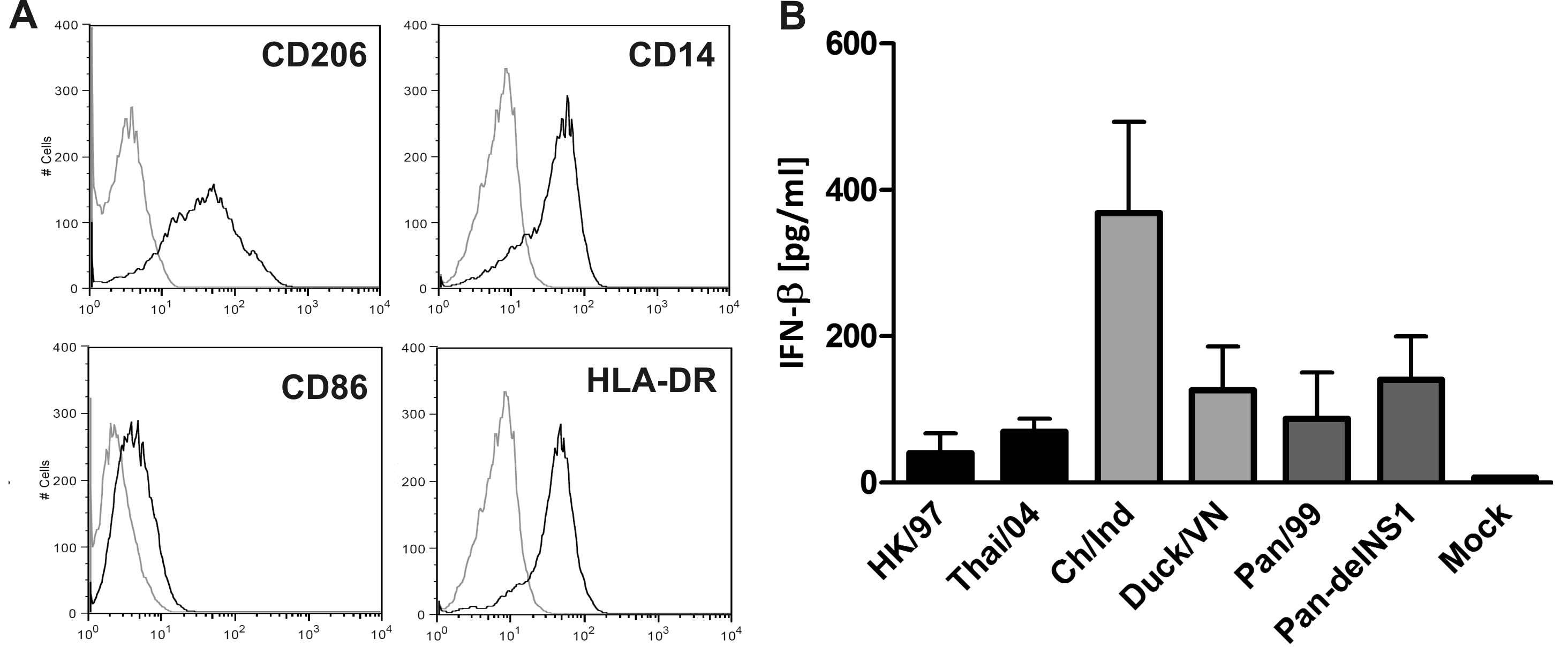

Supplement: Figure S2 — IFN-β secretion of influenza virus-infected human monocyte derived macrophages. (A) Monocytic cells were isolated from buffy coats of healthy human blood donors and were differentiated in vitro. The resulting cultured cells are shown to express the typical markers for macrophages via antibody staining and FACS analysis. The prominent population of cells in the forward/sideward scattering (>90% of the cells, data not shown) expresses CD206, CD14, HLA-DR and little CD86. Black lines indicate the number of cells with a specific signal intensity of the used antibodies, grey lines represent isotype antibody controls. (B) Monocyte-derived human macrophages were infected for 24 h (MOI = 2) with the human and avian H5N1 strains, Pan/99 (H3N2) and its mutant variant with a deleted NS1 gene. IFN-β concentrations of cell culture supernatants were determined via a bead-based cytokine assay (Panomics). Shown are mean IFN-β concentrations +/− SEM of macrophage cultures obtained from three different donors independently infected in triplicates. (TIF) [file pone.0056659.s002.tif]
