# Supplementary material for: Highly Pathogenic H5N1 Influenza A Virus Strains Provoke Heterogeneous IFN-α/β Responses That Distinctively Affect Viral Propagation in Human Cells
Source: PLoS One. 2013 Feb 25;8(2):e56659. doi: 10.1371/journal.pone.0056659 (PMC3581526; doi:10.1371/journal.pone.0056659)
Supplement: Methods S1 — Preparation and infection of monocyte-derived macrophages and FACS analysis. (DOCX) [file pone.0056659.s003.docx]

## ****Supplementary methods****

## ****Preparation and infection of monocyte-derived macrophages and FACS analysis.****

Buffy coats of healthy human donors obtained *via* the German Red Cross were diluted 1:1 in RPMI1640 (Invitrogen) containing 5% FBS and 0.2 mM EDTA and separated by centrifugation with LSM 1077 medium (PAA). The buffy coat fraction was washed and further separated by centrifugation with LSM 1077 diluted with 15% PBS. The resulting cell fraction was washed once in PBS and seeded into 24 wells (3x10**^6^** cells/well) for 2 h. The non-adhered cells were removed and the adherent cells were cultured for 10 days in RPMI1640 supplemented with 10% FBS and 4 mM L-glutamine. The resulting cultured cells were verified by fluorescence activated cell sorting (FACS) analysis to express CD206, CD14 and HLA-DR and to be negative for markers of other immune cells (CD3, CD56, CD80, and CD154) using corresponding fluorophore-labeled antibodies (BD Pharmingen). This analysis was conducted with a FACSCalibur cytometer and the data was analyzed with CellQuest software (BD Biosciences). The used macrophage cultures contained more than 90% CD206 and CD14 marker antigen expressing cells (Fig. S2A). Macrophage cultures of three donors were separately infected in triplicates at a MOI of 2 and incubated for 24 hrs in RPMI1640/ 0.2% BA. Subsequently, cell culture supernatants were UV-inactivated and the concentration of IFN-β was determined on a Luminex 100 system using an IFN-β-specific fluorescence-bead based ELISA (Panomics) according to the manufacturer’s instructions.
